# Prediction model for pneumonia in primary care patients with an acute respiratory tract infection: role of symptoms, signs, and biomarkers

**DOI:** 10.1186/s12879-019-4611-1

**Published:** 2019-11-20

**Authors:** G. H. Groeneveld, J. W. van ’t Wout, N. J. Aarts, C. J. van Rooden, T. J. M. Verheij, C. M. Cobbaert, E. J. Kuijper, J. J. C. de Vries, J. T. van Dissel

**Affiliations:** 1 Department of Internal Medicine and Infectious Diseases, Leiden University Medical Center, P.O. box 9600, 2300 RC Leiden, the Netherlands; 2 Department of Radiology, HMC Bronovo, P.O. box 432, 2501 CK The Hague, the Netherlands; 3 Department of Radiology, HAGA hospital, P.O. box 40551, 2504 LN The Hague, the Netherlands; 4 Julius Center for Health Sciences and Primary Care, University Medical Center Utrecht, PO Box 85500, 3508 GA Utrecht, the Netherlands; 5 Department of Clinical Chemistry and Laboratory Medicine, Leiden University Medical Center, P.O. box 9600, 2300 RC Leiden, the Netherlands; 6 Department of Medical Microbiology, Leiden University Medical Center, Leiden, P.O. box 9600, 2300 RC Leiden, the Netherlands; 7 Centre for Infectious Disease Control, National Institute for Public Health and the Environment (Rijksinstituut voor Volksgezondheid en Milieu, RIVM), Bilthoven, the Netherlands; 8 Department of infectious diseases, Leiden University Medical Center, Leiden, the Netherlands

**Keywords:** Respiratory tract infection, Pneumonia, Primary care, Biomarkers, Prediction model, CRP, Antibiotic, Chest X ray

## Abstract

**Background:**

Diagnosing pneumonia can be challenging in general practice but is essential to distinguish from other respiratory tract infections because of treatment choice and outcome prediction. We determined predictive signs, symptoms and biomarkers for the presence of pneumonia in patients with acute respiratory tract infection in primary care.

**Methods:**

From March 2012 until May 2016 we did a prospective observational cohort study in three radiology departments in the Leiden-The Hague area, The Netherlands. From adult patients we collected clinical characteristics and biomarkers, chest X ray results and outcome. To assess the predictive value of C-reactive protein (CRP), procalcitonin and midregional pro-adrenomedullin for pneumonia, univariate and multivariate binary logistic regression were used to determine risk factors and to develop a prediction model.

**Results:**

Two hundred forty-nine patients were included of whom 30 (12%) displayed a consolidation on chest X ray. Absence of runny nose and whether or not a patient felt ill were independent predictors for pneumonia. CRP predicts pneumonia better than the other biomarkers but adding CRP to the clinical model did not improve classification (− 4%); however, CRP helped guidance of the decision which patients should be given antibiotics.

**Conclusions:**

Adding CRP measurements to a clinical model in selected patients with an acute respiratory infection does not improve prediction of pneumonia, but does help in giving guidance on which patients to treat with antibiotics. Our findings put the use of biomarkers and chest X ray in diagnosing pneumonia and for treatment decisions into some perspective for general practitioners.

## Background

Diagnosing pneumonia in general practice can be challenging. The recognition of pneumonia among other manifestations of respiratory tract infection (RTI) is important since pneumonia – according to the GP’s guideline – requires antimicrobial treatment, has a worse prognosis than other RTIs and requires follow up. Pneumonia comprises (typical and atypical) bacterial and viral infection; the latter is not expected to benefit from antibacterial treatment. On the contrary, acute bronchitis and upper respiratory tract infections are most often of viral origin, and have an excellent prognosis and expectant strategy is generally appropriate [1–3]. To differentiate pneumonia from other respiratory tract infections, clues to determine this diagnosis are needed. Unfortunately, anamnesis and physical examination lack sensitivity and specificity to diagnose pneumonia [4]. Severely ill patients are more likely to have pneumonia, with a high pre-chance of bacterial origin, and should be treated with antibiotics while patients with uncomplicated respiratory tract infection are less ill and have no benefit from being treated with antibiotics. C-reactive protein (CRP) can help to confirm or rule out pneumonia, taking clinical signs and symptoms into account [5]. In particular for moderately ill patients, different guidelines (e.g. the Dutch and the British guideline) point to the use of the CRP test. A low CRP (< 20 mg/l) can rule out pneumonia with reasonable certainty, irrespective of clinical signs and symptoms, while an elevated CRP level (> 100 mg/l) increases the chance of pneumonia and indicates a potential benefit from antibiotic treatment [6, 7]. With CRP levels between 20 and 100 mg/l, decision to initiate antibiotics is left to the clinical picture and assessment of risk factors for a worse outcome [8, 9]. The impact of this strategy on antibiotic prescription rate showed variable results [10].

Among other biomarkers for inflammation, procalcitonin (PCT) had limited added value in the diagnosis of pneumonia in this setting and studies on the prognostic value of the adrenomedullin precursor, mid-regional pro-adrenomedullin (MR-proADM), are currently lacking [7].

The reference ‘golden’ standard for establishing pneumonia is the chest X ray. A chest X ray in outpatients, however, does not improve outcome [11, 12] and therefore this is not routinely recommended in patients attending their general practitioner (GP) with suspicion of a community-acquired pneumonia (CAP). Different general practice guidelines do not provide clear guidance when to order a chest X ray in specific patients with acute respiratory infections [9, 13]. Despite that, in 22% of patients with a suspected lower respiratory tract infection chest X ray is requested [14].

A survey among 255 Dutch GPs in 2014 learned that there is an urgent clinical need for an algorithm to define which patients with an acute respiratory tract infection benefit most from a diagnostic chest X ray [15]. In the Netherlands, general practitioners ordered 31 chest X rays per 1000 persons per year in 2000 [16]. A large proportion of these are intended for patients with acute respiratory tract infections.

Herein, we evaluate a cohort of patients with an acute respiratory tract infection who had been referred by their GP for a chest X ray, to determine predictive factors for the presence of pneumonia.

## Methods

From March 2012 until May 2016 we did a prospective observational cohort study in three radiology departments in different hospitals in the western part of the Netherlands. Local ethical committee approved the study (protocol no. P08.065) and all participants provided written informed consent.

We included adult patients with an acute respiratory tract infection, referred to the radiology department by their general practitioner for a chest X ray to determine the presence of pneumonia. We confined the study to those patients with complaints for less than 3 weeks, as we intended to study the value in ‘acute respiratory tract infection’.

Within an hour before or after chest X ray, clinical data were recorded via an interview and vital signs were measured. Diagnostic tests to find the causal agent of respiratory tract infection were taken: blood cultures were drawn, nasopharyngeal swabs for respiratory viruses and Mycoplasma, Chlamydia and Legionella spp. were collected, a sputum culture (to identify bacterial respiratory pathogens) was taken from persons who coughed up sputum. Blood samples were taken for biomarker testing. At inclusion, EDTA plasma was collected to determine CRP, PCT and MR-proADM. CRP is measured quantitatively with a turbidimetric reaction detecting antigen-antibody complex (Roche Modular P800) (catalogue number 12000951/12000953/04956923190).

PCT is measured with Brahms Kryptor using an immunoassay with TRACE (Time Resolved Amplified Cryptate Emission) technology (catalogue number 82591/82592/825050).

MR-proADM is measured with Brahms Kryptor with an automated immunofluorescence assay using TRACE technology (catalogue number 82991/82992/829050).

Chest X-ray was made by GP’s request and was not part of the study protocol. Both postero-anterior and lateral view were obtained. Radiology reports were made by certified radiologists with no knowledge of the current study. For an individual patient, one radiologist made a written report, with a clear conclusion, as part of regular patient care. These reports, intended for the GPs, were used to determine whether or not a consolidation was present.

We did not intervene with the GP’s treatment strategy.

After 30 days, a follow up contact via telephone call was made. In this standardized telephone interview, clinical symptoms were evaluated, any antibiotic usage documented, and resolution of symptoms and newly diagnosed disease entities noted.

Our primary end point was the presence of a consolidation on chest X ray, i.e. pneumonia. In the past, several models with clinical signs and symptoms with or without biomarkers (CRP, PCT and MR-proADM) have been used to predict pneumonia [5, 7]. With these models we compared the ability of biomarkers to correctly improve a prediction versus the situation where biomarkers are not available.

For prediction of pneumonia we used three predefined diagnostic risk groups, assessing the probability of the presence of pneumonia on chest X ray. These risk groups should facilitate clinical decision making, e.g. starting antibiotic treatment. We anticipated that these risk groups would be used in clinical practice with signs and symptoms and to assess the added value of biomarkers. We defined a low risk group with a probability of pneumonia less than 2.5%, an intermediate risk group with a probability of pneumonia between 2.5 and 20% and a high risk group with a probability of pneumonia above 20%. We have chosen these cut-off values of the risk groups as these roughly represent daily decision making in general practice. With these cut-off values safe clinical decision making is possible in daily practice. Comparable risk groups have been used in the GRACE cohort [7].

Predictors for pneumonia were selected using multivariate regression models. With equations derived from the multivariate regression models without and with biomarkers, we could identify patients in low, intermediate and high risk groups of pneumonia.

As only low and high risk of pneumonia would have clear consequences for GP management, i.e. withholding or prescribing antibiotic treatment respectively, we pose that change to a higher risk group in cases with pneumonia and to a lower risk group in cases without pneumonia would reflect useful reclassification which could improve decision making.

To calculate the overall reclassification improvement, we subtracted patients who were reclassified incorrectly from those who reclassified correctly and divide this number by the total number of study patients.

Secondary outcome measures were the presence of bacterial or viral agents and the antibiotic courses used in patients with and without pneumonia and in patients with or without bacterial infection. CRP, PCT and MR-proADM values were evaluated for their predictive ability for pneumonia, 30-day mortality and need for secondary care. We evaluated antibiotic courses in patients with treatable disease, i.e. consolidation or bacterial pathogen detected.

We also used our data to evaluate the findings of the GRACE cohort. Our findings were entered in the multivariate model of the GRACE cohort to assess the value of biomarkers to improve prediction by calculating the overall reclassification improvement.

Will their strategy to predict consolidation on chest X-ray (i.e. pneumonia) apply in our cohort? The results of this evaluation are described in the supplementary material (section ‘The GRACE analysis in the current study cohort’ including Additional file 1: Table S5A-C).

### Statistics

We used descriptive statistics to describe baseline characteristics. Descriptive analysis included means with confidence intervals or medians and interquartile ranges, as appropriate.

To assess the predictive value of CRP, PCT and MR-proADM for pneumonia, area-under-the-curve (AUC) of receiver operating characteristics (ROC) curves were calculated. This analysis determined which biomarker will be used in the regression model.

Univariate and multivariate binary logistic regression will be used to evaluate clinical parameters and biomarkers (CRP, PCT and MR-proADM) as predictors for pneumonia. The multivariate prediction model of our cohort consists of variables which are clinically relevant or have a *P* value less than 0.1 in univariate analysis.

Cut off points for CRP and PCT as they have been used in the GRACE algorithm will be used. For MR-proADM two cut off points will be used. The first MR-proADM cut off point is 0.646 nmol/l. This was the optimal cut off point to discriminate patients with low risk community acquired pneumonia (pneumonia severity index, PSI, I-III) from patients with high risk CAP (PSI IV and V) with sensitivity of 92% and specificity of 55% [17].

The second MR-proADM cut off point is 1.00 nmol/l. In patients with febrile urinary tract infections, this is the optimal cut off to predict 30 day mortality [18].

## Results

Between March 2012 and March 2016 we included 249 patients via alternating radiology departments from 2 teaching hospitals and 1 regional hospital in the western part of the Netherlands. The patients were included during all seasons of the year. Baseline characteristics of the cohort are described in Table 1 and in the (Additional file 1: Table S1).

**Table 1:** Baseline characteristics of the cohort

Total number of patients	249
Female (%)	127 (51.0)
Median age in years (IQR)	56 (43–67)
Duration of complaints:	
- Less than a week (%)	45 (18.1)
- Between 1 and 2 weeks (%)	104 (41.8)
- Between 2 and 3 weeks (%)	97 (39.0)
Comorbidity (%)	196 (78.7)
Hospital admission in previous year (%)	32 (12.9)
Received influenza vaccination (%)	108 (43.4)
Antibiotic usage previous 3 months (%)	
- None	121 (48.6)
- One course	95 (38.2)
- More than one course	33 (13.3)
Antibiotic courses (%)	
- Amoxicillin	48 (29.6)
- Amoxicillin with clavulanic acid	7 (4.3)
- Penicillin	5 (3.1)
- Doxycycline	38 (23.5)
- Macrolide	14 (8.6)
- Quinolone	3 (1,9)
- Other	4 (2.5)
- Unknown antibiotic	43 (26.5)
Smoking (previous or current) (%)	141 (56,6)
Median CRB-65 score^a^ (IQR)	0 (0–1)

*IQR* interquartile range

^a^CRB-65 severity score predicting 30 day mortality with higher score implicating higher 30 day mortality. C = new onset confusion, R = respiratory rate ≥ 30/min, B = Blood pressure (Systolic < 90 mmHg or Diastolic ≤60 mmHg), 65 = Age ≥ 65

### Detection of pneumonia on chest X ray

In 30 (12%) of patients, a pneumonia was detected on chest X ray.

### Detection of respiratory pathogen as cause of infection

In our study, in 41% of patients a viral infection was established, in 1% a pneumococcal infection, in 2% a *Haemophilus influenzae* infection. In two patients *Mycoplasma pneumoniae* (in sputum) was detected, in three patients (two sputum samples and one nasopharyngeal swab) *Chlamydia pneumoniae* and in three sputum samples *Legionella spp.* was detected (*Legionella pneumophila* PCR was negative in these patients). Respectively one (3.7%), three (11.1%) and two (7.4%) had a consolidation on chest X ray (see Additional file 1: Table S2 in the supplementary appendix). In one of the eight patients with an atypical pathogen (i.e. *Legionella spp.*), both *S. pneumoniae* and rhinovirus were detected.

### Antibiotic prescriptions

A total number of 104 antibiotics were prescribed for 83 patients (Additional file 1: Table S1). Of all patients with consolidation or bacterial pathogen detected (treatable disease), 19/33 (58%) have received one or more antibiotic courses after chest X ray. Of 199 patients without treatable disease, 64 (32%) have received antibiotic treatment.

Thirty-six patients (14%) were referred to the hospital (24 outpatient clinic and 12 were admitted), none of the patients died within 30 days after chest X ray*.* Neither CRP nor PCT nor MR-proADM could predict the need for hospital care within 30 days after chest X ray (Additional file 1: Figure S1 ROC curve biomarkers and need for hospital care after chest X ray).

In two patients, abnormalities besides consolidation were detected. During follow up, the first appeared to be a calcified benign nodus and the second appeared to be atelectasis due to a mucus plug. No malignancies were detected. More outcome details are available in Additional file 1: Table S1.

### Prediction for pneumonia

Univariate analysis of clinical risk factors for pneumonia is described in Additional file 1: Table S3. Antibiotic use in the previous 3 months or influenza vaccination was not a risk factor for pneumonia in our cohort. We drafted three age cohorts with the same number of patients in each cohort (eight patients aged 64 were present, these were all categorised in the eldest group).

Results of multivariate analysis with signs and symptoms are described in Table 2. Absence of runny nose and whether or not a patient felt ill were independent predictors for pneumonia in our clinical risk model. Calibration of this model was good with a Hosmer-Lemeshow test of 4.53 (df = 7, *P* = 0.72); Nagelkerke R square 0.29.

**Table 2:** Multivariate analysis of clinical variables in prediction model for pneumonia in 249 patients presenting at radiology department with acute respiratory tract infection in primary care

Diagnostic variable	Multivariable OR (95%CI)	*P* value
Age cohort (18–47 years is reference category)		0.28
• 48–63	2.17 (0.65–7.26)	
• ≥64	0.49 (0.03–7.44)	
Runny nose absent	3.00 (1.23–7.33)	0.02
Feel ill	14.89 (3.27–67.91)	0.00
Current smoker	0.34 (0.09–1.39)	0.13
Oxygen saturation	0.88 (0.67–1.16)	0.38
CRB-65 score^a^ (0 is reference category) • 1	6.77 (0.51–89.78)	0.35

^a^CRB-65 severity score predicting 30 day mortality with higher score implicating higher 30 day mortality. C = new onset confusion, R = respiratory rate ≥ 30/min, B = Blood pressure (Systolic < 90 mmHg or Diastolic ≤60 mmHg), 65 = Age ≥ 65

No values for CRB65 score of 2 since only 2 patients were present in that category

With variables in multivariate analysis with *P* < 0.10, we made the prediction equation (see Additional file 1: Table S4A in the supplementary appendix) for the presence of a consolidation on chest X ray, using clinical signs and symptoms only:

$$ 1/\left(1+\exp -\left(-4.492+1.142\times \mathrm{absence}\ \mathrm{of}\ \mathrm{runny}\ \mathrm{nose}\left(0\mathrm{or}1\right)+2.550\times \mathrm{feel}\ \mathrm{ill}\left(0\mathrm{or}1\right)\right)\right) $$

### Biomarker for guidance of the presence of pneumonia

In Table 3 multivariate analysis of clinical variables and biomarkers predicting pneumonia are described. Calibration of this model was good with a Hosmer-Lemeshow test of 10.09 (df = 8, *P* = 0.26); Nagelkerke R square 0.36.

**Table 3:** Multivariate analysis of clinical variables and biomarkers in prediction model for pneumonia in 249 patients presenting at radiology department with acute respiratory tract infection in primary care

Diagnostic variable	Multivariable OR (95%CI)	*P* value
Age cohort (18–47 years is reference category)		0.54
• 48–63	1.74 (0.48–6.30)	
• ≥64	0.61 (0.05–8.07)	
Runny nose absent	3.12 (1.22–8.00)	0.02
Feel ill	13.33 (2.80–63.40)	0.00
Current smoker	0.27 (0.06–1.19)	0.08
Oxygen saturation	0.94 (0.72–1.24)	0.68
CRB-65 score^a^ (0 IS REFERENCE CATEGORY)		0.41
• 1	5.29 (0.46–61.15)	
CRP > 30 mg/l	4.66 (1.73–12.55)	0.00

^a^CRB-65 severity score predicting 30 day mortality with higher score implicating higher 30 day mortality. C = new onset confusion, R = respiratory rate ≥ 30/min, B = Blood pressure (Systolic < 90 mmHg or Diastolic ≤60 mmHg), 65 = Age ≥ 65

No values for CRB65 score of 2 since only 2 patients were present in that category

In univariate analysis, CRP predicts pneumonia better than PCT and MR-proADM do (Additional file 1: Table S3 and Figure S2. ROC curve biomarkers and pneumonia on chest X ray). Therefore, only CRP is present in the multivariate model. We have used the cut-off point of 30 mg/l to make the results comparable with the GRACE findings.

With variables in multivariate analysis which are clinically relevant or have *P* < 0.10 (we did not use current smoker since this represents more likely the type of patients for which chest X ray was deemed necessary), we made the prediction equation (see Additional file 1: Table S4B in the supplementary appendix) for the presence of a consolidation on chest X ray, using clinical signs and symptoms and CRP (> 30 mg/l):

$$ 1/\left(1+\exp -\left(-4.797+1.230\times \mathrm{absence}\ \mathrm{of}\ \mathrm{runny}\ \mathrm{nose}\left(0\mathrm{or}1\right)+2.378\times \mathrm{feel}\ \mathrm{ill}\left(0\mathrm{or}1\right)+1.572\times \mathrm{CRP}>30\mathrm{mg}/\mathrm{l}\left(0\mathrm{or}1\right)\right)\right) $$

In Table 4, the reclassification with CRP added to the model is described. The improvement in classification can now be calculated. Of all patients with pneumonia, 8 are reclassified to higher risk group and 0 to lower risk groups. Reclassification improvement in patients with pneumonia is 8/30 = 26.7%.

**Table 4:** Reclassification table using results from multivariate analysis

Risk according to sign and symptoms without CRP	Risk according to signs and symptoms plus CRP > 30 mg/l							
	Patients with pneumonia				Patients without pneumonia			
	<2.5%	2.5–20%	> 20%	Total	<2.5%	2.5–20%	> 20%	Total
<2.5%	0	0	0	0	49	2	0	51
2.5–20%	0	8	8	16	0	115	15	130
> 20%	0	0	14	14	0	0	31	31
Total	0	8	22	30	49	117	46	212

In 1 patient clinical variable is missing; in 6 patients CR*P* value is missing

In patients without pneumonia reclassification improvement is (0–17)/212 = − 8.0%. From the total cohort, 8 have been reclassified correctly and 17 have been reclassified incorrectly. Therefore, the overall reclassification improvement is − 9/242 = − 3.7% with adding CRP to the model.

Twenty-three patients (16%) in the intermediate risk group with signs and symptoms only, were reclassified into high risk group when adding CRP to the model. Eight of these (35%) had pneumonia. None were reclassified into low risk group.

Using our own model with CRP, consolidation was present in none in the low risk group, 6.4% in the intermediate risk group and 32.4% in the high risk group.

## Discussion

In patients referred by their general practitioner for a chest X-ray in the course of an acute respiratory tract infection, one in eight (12%) showed a consolidation on the chest X ray, i.e., was diagnosed with community acquired pneumonia. Biomarkers like CRP, PCT and MR-proADM do not help discriminate between presence or absence of an infiltrate on chest X-ray over that of a model with clinical characteristics only, but CRP did help to guide the physician on treatment decisions.

In all low risk patients (21% of study population) a pneumonia is absent and therefore, the chest X ray has very little added value.

Our study has several strong and weak points. Strengths of our study are the fairly complete patient data including 30 day follow up and the extensive microbiological testing. During the study project, we found that none of the patients had positive blood cultures; therefore, because of futility, we stopped collecting blood cultures after the first 92 blood cultures proved negative.

The findings in our study underscore the importance of collecting some basic patient data in daily primary care. For instance, the question about the patient feeling ill proved to be the best independent predictor for the presence of pneumonia in our cohort. This is in line with other reports [19, 20].

Latest report about aetiology in CAP in the Netherlands stems from 2004; in that report, 10% of the patients was infected with an atypical pathogen [21].

Since 2011, the Dutch guideline ‘Acute cough’ prescribes to start with amoxicillin antibiotic treatment instead of doxycycline in patients with presumptive pneumonia [9]. Apparently, this change in empiric treatment has not resulted in an increased prevalence of atypical pathogens in those who present with persistent cough despite amoxicillin therapy. In our cohort the prevalence of atypical pathogens was 4% only and in many cases it remained uncertain whether these pathogens were the cause of infection or represent asymptomatic carriage [22]. Interestingly, 20% of patients with consolidation on X-ray in our study had microbiological proven *Legionella spp.*, *Mycoplasma pneumoniae* or *Chlamydia pneumoniae*.

We used nasopharyngeal swabs for virus and atypical pathogen detection. Although sputum samples have a higher detection rate than nasopharyngeal swabs, adequate sputum samples were only available in a minority of patients and they were used to culture bacterial pathogens [23]. With more adequate sputum samples available and with both culture and molecular testing on these sputum samples, diagnostic yield might have been increased. In addition, prolonged illness or prolonged coughing is a frequent symptom after clearance of the causative agent in respiratory tract infection [24, 25]. Presumably, in a proportion of patients the causative agent has already been cleared while symptoms are still present.

Collection of patient data, diagnostic sampling and chest X ray were all within 1 hour. Therefore, all our results reflect the same stage of disease.

Another strength is the value of our cohort to evaluate the GRACE findings in a different cohort of patients (see supplementary material).

Although, there are several weaknesses in our study that need explanation. Since we did not include the patients at the general practice, we do not have the results of physical examination of the GP (crackles and diminished vesicular breathing). In the GRACE study these variables were important in predicting pneumonia.

We chose to include patients with a chest X ray since this examination is considered the gold standard for the presence or absence of pneumonia. Therefore, we have included patients at radiology departments. In the Netherlands, general practitioners do not have their own radiology facilities at their practice. Primary care patients should visit a hospital for a chest X ray making this diagnostic a demanding procedure for patients. Our study includes a selected proportion of patients with an acute respiratory tract infection. In these study patients, GPs felt the patient might benefit from a chest X ray as it would confirm or refute a pneumonia or other lung pathology. This is a small fraction of the total number of patients visiting their GP with and acute respiratory tract infection [15]. The patients who were not referred for chest X ray were diagnosed and treated according to the Dutch guideline ‘Acute Cough’, and GPs estimated that these patients would not benefit from a chest X ray, as these did not present a diagnostic dilemma [9].

This selection results in a study population of patients who have not responded to GP’s empirical therapy, patient for whom doubt about diagnosis or treatment is present or – as assessed by the GP – have a high chance of showing other relevant pulmonary abnormalities. Almost 80% of the study patients had co-morbidity, more than 50% has used antibiotics in the previous 3 months and 81% of patients had complaints for more than 1 week. The majority of patients did not show a pneumonia (219/249) and had only mild disease given their median CRB-65 score of 0 (IQR 0–1). The results of this study are therefore generalisable to this specific patient population. The finding that current smoking is negatively associated with the presence of pneumonia, suggests that GPs have lower threshold to order chest X ray in smoking than in non-smoking patients (Additional file 1: Table S3).

Although at first site it seems counterintuitive that patients aged 48–63 years are at increased risk for having pneumonia and older patients have relative low risk (Table 3), it is highly likely that older patients are referred for chest X ray earlier than younger patients.

Although we included patients year-round and the GRACE study included patients during winter months, the percentage of patients having pneumonia in our cohort (12%) is considerably higher (12 versus 5%). This is to be expected since these patients were selected by GPs assessment to be at risk for CAP or another serious lung disorder [26]. The proportion of patients with pneumonia in our cohort corresponds with the numbers found in other cohorts [6, 27].

The GRACE model was developed to help GPs in the decision regarding the diagnosis of patients with acute cough. Our study includes a selected proportion of patients with acute cough and therefore (15) our cohort is enriched with patients with pneumonia (12%) compared to the GRACE cohort (5%). In addition, only a minority of consolidations has disappeared on chest X ray in the first 3 weeks after start of treatment of pneumonia [28, 29]. Therefore, we suppose that the consolidations present at start of complaints, would still be visible on chest X ray during our study.

Overall reclassification improvement in the GRACE model was 29% [7]. In this mildly ill cohort, the reclassification improvement was mainly due to reclassifying patients from intermediate risk to the low risk group.

On the contrary, in our study cohort of more severely ill patients, most benefit was present in reclassifying intermediate risk patients to the high risk group. On the basis of a CRP measurement, 23 of 146 patients (16%) in the intermediate risk group should be reclassified into the high risk group. Because this group is enriched for persons with pneumonia, it would likely benefit from antibiotic treatment. However, CRP did not help in reclassification of intermediates into the low risk group.

Reclassification from the intermediate risk group into the low or high risk group, by adding CRP level to the diagnostic process but without a chest X ray, would be relevant in daily practice. Also classification into low or high risk group would have direct impact on treatment decision, respectively withhold or initiate antibiotic treatment, and these results could have implications for future decision making in general practice. In our model using CRP (Table 4), 117/242 (48%) is classified in either the low or the high risk group. Thus, for these patients, an antibiotic treatment decision can be made without a chest X ray. These findings need to be validated in a new cohort.

We have chosen to use overall reclassification improvement instead of net reclassification improvement as it was used in the GRACE analysis [7]. The net reclassification counts the percentages of two groups (with and without pneumonia), with complete different numbers of patients (30 patients with pneumonia versus 212 without). This leads to overrepresentation of the percentage from the smallest group of patients. The overall reclassification improvement values every patient in the same way, with or without pneumonia.

The overall reclassification improvement with CRP added to the model, did not help to discriminate between presence or absence of an infiltrate on chest X-ray over that of a model with clinical characteristics only (− 3.7%). CRP did help to guide the physician on treatment decisions since 23 patients (Table 4) were reclassified into the high risk group that – according to guidelines – warrant antibiotic treatment. Eight of these 23 reclassified patients (35%) had pneumonia.

Using our model for antibiotic treatment decision in patients for whom chest X ray is considered during acute respiratory tract infection, clinical signs and symptoms alone can identify patients at low risk for pneumonia (who should not be treated) and patients a high risk for pneumonia who probably benefit from antibiotic treatment. Patients who are at intermediate risk (2,5–20%) for having pneumonia, using clinical signs and symptoms only, would benefit from CRP testing to identify the patients who have a high risk of pneumonia. Of 146 intermediate risk patients, 23 (16%) would be reclassified in the high risk group when adding CRP in the decision model (Table 4).

In general the different kinetics such as a short half-life, especially for MR-proADM, make markers like PCT and MR-proADM of less value than CRP when it comes to diagnose and treat pneumonia in general practice. Also, MR-proADM is released from endothelium in response to systemic inflammation and is a marker of severity of pneumonia [30]. Our cohort of primary care patients, however, displayed little systemic inflammation and this may have deemed MR-proADM less clinically relevant. Studies on MR-proADM as a biomarker in respiratory tract infections in primary care are scarce [31]. Our findings of CRP and procalcitonin are in accordance with other studies [7, 32].

## Conclusions

Our model would preclude the need for a diagnostic chest X rays in 21% of GP patients with an acute respiratory tract infection (the low risk group). CRP predicts pneumonia better than the other biomarkers but adding CRP to the clinical model did not improve classification (− 4%); however, CRP helped guidance of the decision which patients should be given antibiotics. Our findings put the use of biomarkers and chest X ray in diagnosing pneumonia and for treatment decisions into some perspective for general practitioners.

## Supplementary information

**Additional file 1.** Supplementary material ‘Prediction model for pneumonia in primary care patients with an acute respiratory tract infection: role of symptoms, signs, and biomarkers’.

## Data Availability

The datasets generated during and/or analysed during the current study are available from the corresponding author on reasonable request.

## Abbreviations

AUC: area-under-the-curve
CAP: community-acquired pneumonia
CRP: C-reactive protein
GP: general practitioner
IQR: interquartile range
MR-proADM: mid-regional pro-adrenomedullin
PCT: procalcitonin
PSI: pneumonia severity index
ROC curve: receiver operating characteristics curve
RTI: respiratory tract infection
TRACE technology: Time Resolved Amplified Cryptate Emission technology
