# Supplementary material for: Prediction model for pneumonia in primary care patients with an acute respiratory tract infection: role of symptoms, signs, and biomarkers
Source: BMC Infect Dis. 2019 Nov 20;19:976. doi: 10.1186/s12879-019-4611-1 (PMC6865035; doi:10.1186/s12879-019-4611-1)
Supplement: Supplementary file 1 — Additional file 1. Supplementary material ‘Prediction model for pneumonia in primary care patients with an acute respiratory tract infection: role of symptoms, signs, and biomarkers’. [file 12879_2019_4611_MOESM1_ESM.docx]

**Supplementary material ‘Prediction model for pneumonia in primary care patients with an acute respiratory tract infection: role of symptoms, signs, and biomarkers’**

Table S1. Characteristics of the cohort

| **Baseline characteristics** | |
| --- | --- |
| Total number of patients | 249 |
| Female (%) | 127 (51.0) |
| Median age in years (Interquartile range) | 56 (43-67) |
| Duration of complaints:   - Less than a week (%) - Between one and two weeks (%) - Between two and three weeks (%) | 45 (18.1)  104 (41.8)  97 (39.0) |
| Throat pain (%) | 86 (34.5) |
| Coryza/runny nose (%) | 159 (63.9) |
| Cough (%) | 223 (89.6) |
| Sputum (%) | 185 (74.3) |
| Dyspnoea (%) | 189 (75.9) |
| Fever or feverishness (%) | 173 (69.5) |
| Days since the last episode of fever(%):   - 0, today fever - 1, yesterday fever - 2 days ago fever - More than 2 days ago fever - Not applicable or don’t know | 21 (8.5)  21 (8.5)  10 (4.0) 71 (28.6)  125 (50.4) |
| Myalgia (%) | 101 (40.6) |
| Headache (%) | 125 (50.2) |
| Joint pain (%) | 55 (22.1) |
| Feel ill (%) | 155 (62.2) |
| Comorbidity (%) | 196 (78.7) |
| Hospital admission in previous year (%) | 32 (12.9) |
| Most recent hospital discharge:   - 0-3 months ago - 4-6 months ago - 6-12 months ago | 10  5  17 |
| Visited foreign country in previous 3 months (%) | 82 (32.9) |
| Received invitation for influenza vaccination (%) | 155 (62.2) |
| Received influenza vaccination (%) | 108 (43.4) |
| Antibiotic usage previous 3 months (%)   - None - One course - More than one course | 121 (48.6)  95 (38.2)  33 (13.3) |
| Antibiotic courses (%)   - Amoxicillin - Amoxicillin with clavulanic acid - Penicillin - Doxycycline - Macrolide - Quinolone - Other - Unknown antibiotic | 48 (29.6) 7 (4.3)  5 (3.1)  38 (23.5)  14 (8.6)  3 (1,9)  4 (2.5)  43 (26.5) |
| ADL* support (%) | 7 (2.8) |
| Pregnant or breastfeeding (%) | 0 (0) |
| Smoking (previous or current) (%)   - Median packyears of those who have current or previous smoking (interquartile range) | 141 (56.6)  19 (8-30) |
| Race or ethnic group (%)   - White/Caucasian - Asian - North African - Black | 221 (88.8)  20 (8.0)  1 (0.4)  4 (1.6) |
| Plan after chest X ray   - It will be determined after chest X ray result is available for GP - Start with antibiotics - Start with medical treatment other than antibiotic - There is no plan | 188 (75.5)  42 (16.9)  2 (0.8)  17 (6.8) |
| **Outcome** | |
| 30 day mortality | 0 (0) |
| Outcome day 30   - Complete recovery - Missing | - 147 (59.0) - 17 (6.8) |
| Total duration of complaints   - <1 week - 1-<2 weeks - 2-<3 weeks - ≥3 weeks - Missing | - 5 (2.0) - 29 (11.6) - 46 (18.5) - 152 (61.0) - 17 (6.8) |
| Duration of fever   - 0 - 1-3 - 4-7 - 8-14 - >14 days - Unknown - Missing | - 117 (47.0) - 44 (17.7) - 38 (15.3) - 10 (4.0) - 9 (3.6) - 15 (6.0) - 16 (6.4) |
| Still coughing   - Yes - No - Missing | - 79 (31.7) - 150 (60.2) - 20 (8.0) |
| Duration of coughing   - 0 days - 1-7 days - 8-14 days - 15-21 days - >21 days - Unknown - Missing | - 19 (7.6) - 14 (5.6) - 31 (12.4) - 34 (13.7) - 132 (53.0) - 3 (1.2) - 16 (6.4) |
| Antibiotic treatment after chest X ray   - No - Yes, 1 course - Yes, > 1 course - Missing | - 149 (59.8) - 64 (25.7) - 19 (7.6) - 17 (6.8) |
| If antibiotic used, which one   - Total number of treatments - Amoxicillin - Amoxicillin/clavulanic acid - Feneticillin - Doxycycline - Macrolide - Quinolone - Unknown | - 104 - 25 - 3 - 4 - 23 - 11 - 4 - 34 |
| Side effects   - Total number - Allergic - Diarrhoea - Nausea/vomiting - Yeast infection - Other | - 30 - 2 - 12 - 6 - 3 - 7 |
| Other treatment besides antibiotics   - No - Oral steroids - Lung inhalers - Oseltamivir - Codeine - Other | - 129 (51.8) - 21 (8.4) - 57 (22.9) - 2 (0.8) - 7 (2.8) - 14 (5.6) |
| Referral to hospital   - No - Outpatient clinic - Admission to ward   - Length of stay     - 1-3 days     - 4-7 days     - 8-14 days     - > 14days | - 196 (78.7) - 24 (9.6) - 12 (4.8) - 6 - 2 - 2 - 2 |

* Activities of Daily Living

Table S2: Outcome details

| Number of patients | **All patients (249)** | **In patients with consolidation on chest X ray (30)** | **In patients without pneumonia (219)** |
| --- | --- | --- | --- |
| Mean age (years) | 55.5 (95% CI 53.5-57.4) | 61.4 (95% CI 55.9-66.8) | 54.7 (95%CI 52.6-56.8) |
| Female | 127 (51.0%) | 13/30 (43.3%) | 114/219 (52.1%) |
| BMI (median) | 26.0 (IQR 23.5-29.6) | 25.3 (IQR 22.9-30.8) | 26.0 (IQR 23.5-29.6) |
| Smoking (history) | 141 (57.8%) | 19/30 (63.3%) | 122/214 (57.0%) |
| Median respiratory rate (median) | 15 (13-18) | 16 (14-20) | 15 (13-18) |
| CRB-65 score 0:1:2 | 170:71:2 | 15:14:0 | 149:58:2 |
| Influenza vaccination | 108 (43.4%) | 15 (50.0%) | 93 (42.5%) |
| Antibiotic pre-treatment | 128 (51.4%) | 19 (63.3%) | 109 (49.8%) |
| **Diagnostic results** | | | |
| Any viral agents | 97/238 | 9/30 (30.0%) | 88/208 (38.6%) |
| Influenza A | 15/237 | 0/30 | 15/207 (6.3%) |
| Influenza B | 5/237 | 0/30 | 5/207 (2.4%) |
| RSV | 3/237 | 0/30 | 3/207 (1.4%) |
| Parainfluenza 1-4 | 10/237 | 0/30 | 10/207 (4.8%) |
| Metapneumovirus | 8/236 | 3/30 (10.0%) | 5/206 (1.7%) |
| Rhinovirus | 47/236 | 4/30 (13.3%) | 43/206 (14.4%) |
| Coronavirus | 7/237 | 2/30 (6.7%) | 5/207 (2.4%) |
| Adenovirus | 2/238 | 0/30 | 2/208 (1.0%) |
| Bocavirus | 0/238 | 0/30 | 0/208 |
| S. pneumoniae | 2/249 | 2/30 (6.7%) | 0/219 |
| H. influenzae | 5/249 | 1/30 | 4/219 |
| H. parainfluenzae | 1/249 | 1/30 | 0/219 |
| K. pneumoniae | 1/249 | 0 | 1/219 |
| Legionella spp. | 3/228 | 2/27 | 1/201 |
| Mycoplasma pneumoniae | 2/228 | 1/27 | 1/228 |
| Chlamydia pneumoniae | 3/228 | 3/27 | 0/228 |
| **After chest X ray** | | | |
| Completely resolved (day 30) | 147/232 (63.4) | 13/26 (50.0) | 134/206 (65.0) |
| Antibiotic use after chest X ray   - No - 1 course - > 1 course | 149/232 (64.2) 64/232 (27.6) 19/232 (8.2) | 8/26 (30.8) 13/26 (50.0) 5/26 (19.2) | 141/206(68.4) 51/206 (24.8) 14/206 (6.8) |
| One or more side effects from antibiotics | 28/122 (23.0) | 8/23 (34.8) | 20/99 (20.2) |
| Lung cancer detected | 0/249 | 0/30 | 0/219 |
| Hospital referral   - No - Outpatient clinic - Admission to ward | 196/232 (84.5) 24/232 (10.3) 12/232 (5.2) | 18/26 (69.2) 4/26 (15.4) 4/26 (15.4) | 178/232 (86.4) 20/206 (9.7) 8/206 (3.9) |
| 30 day mortality | 0/249 | 0/30 | 0/219 |

Table S3. Univariate analysis of diagnostic variables and pneumonia in 249 patients presenting at radiology department with acute respiratory tract infection in primary care.

| Diagnostic variable | Missing | Total (n=249) | Pneumonia present (n=30) | Univariable OR (95%CI) | P value |
| --- | --- | --- | --- | --- | --- |
| Mean (SD) Age (years) | 0 (0.0) | 55 (16) | 61 (15) | 1.03 (1.00-1.06) | 0.03 |
| Age cohort   - 18-47 - 48-63 - ≥64 | 0 | 83  80  86 | 5  11  14 | 2.49 (0.82-7.51)  3.03 (1.04-8.85) | 0.12  0.11  0.04 |
| Men | 0 (0.0) | 122 (49.0) | 17 (57) | 1.42 (0.66-3.06) | 0.37 |
| Current smoker | 5 (2.0) | 58 (24) | 3 (10) | 0.32 (0.09-1.10) | 0.07 |
| Of current smokers:   - Median no. of pack years (IQR) | 6 (10) | 20 (10-30) | 40 (16-43) | 1.02 (0.98-1.07) | 0.36 |
| No. of weeks illness before chest X ray   - < 1 week - 1-2 weeks - >2 weeks and ≤3 weeks | 3 (1.2) | 45 (18) 104 (42) 97 (39) | 9 (30) 10 (33) 11 (37) | 0.43 (0.16-1.13) 0.51 (0.20-1.34) | 0.09  0.17 |
| Cough | 0 (0.0) | 223 (90) | 25 (83) | 0.53 (0.18-1.53) | 0.24 |
| Phlegm | 1 (0.4) | 185 (75) | 19 (66) | 0.61 (0.27-1.39) | 0.24 |
| Breathlessness | 0 (0.0) | 189 (76) | 25 (83) | 1.68 (0.61-4.59) | 0.32 |
| Runny nose absent | 1 (0.4) | 89 (36) | 16 (53) | 2.27 (1.05-4.91) | 0.04 |
| Fever | 0 (0.0) | 173 (70) | 24 (80) | 1.88 (0.74-4.80) | 0.19 |
| Chest pain | 0 (0.0) | 82 (33) | 6 (20) | 0.47 (0.18-1.20) | 0.33 |
| Throat pain | 1 (0.4) | 86 (35) | 13 (43) | 1.52 (0.70-3.30) | 0.29 |
| shivering | 0 (0.0) | 135 (54) | 16 (53) | 0.96 (0.45-2.06) | 0.92 |
| Muscle ache | 0 (0.0) | 101 (41) | 13 (43) | 1.14 (0.53-2.46) | 0.74 |
| Headache | 0 (0.0) | 125 (50) | 17 (57) | 1.34 (0.62-2.90) | 0.45 |
| joint pain | 0 (0.0) | 55 (22) | 7 (23) | 1.08 (0.44-2.68) | 0.86 |
| Feel ill | 0 (0.0) | 155 (62) | 28 (93) | 10.14 (2.36-43.65) | 0.00 |
| Confused | 0 (0.0) | 2 (1) | 1 (3) | 7.52 (0.46-123.46) | 0.16 |
| same symptoms around (thuis + naaste omgeving) | 0 (0.0) | 94 (38) | 9 (30) | 0.68 (0.30-1.55) | 0.35 |
| Birds at home | 0 (0.0) | 20 (8) | 4 (13) | 1.95 (0.61-6.28) | 0.26 |
| Hotel in previous month | 0 (0.0) | 54 (22) | 4 (13) | 0.52 (0.17-1.56) | 0.24 |
| Sauna in previous month | 0 (0.0) | 21 (8) | 2 (7) | 0.75 (0.17-3.40) | 0.71 |
| Received influenza vaccination | 0 (0.0) | 108 (43) | 15 (50) | 1.36 (0.63-2.91) | 0.44 |
| Hospital admission in previous year | 0 (0.0) | 32 (13) | 3 (10) | 0.73 (0.21-2.55) | 0.62 |
| Antibiotic use in previous 3 months | 0 (0.0) | 128 (51) | 19 (63) | 1.74 (0.79-3.84) | 0.17 |
| Any comorbidity (pulmonary, cardiac, diabetes mellitus) | 0 (0.0) | 76 (31) | 11 (37) | 1.37 (0.62-3.04) | 0.44 |
| median syst blood pressure (IQR) | 2 (0.8) | 130 (120-140) | 128 (118-142) | 1.00 (0.97-1.02) | 0.79 |
| Median Diastolic blood pressure (IQR) | 2 (0.8) | 82 (78-90) | 80 (74-90) | 0.98 (0.94-1.02) | 0.34 |
| Tachycardia >100 beats/min | 0 (0.0) | 4 (2) | 1 (3) | 2.48 (0.25-24.67) | 0.44 |
| Temperature >37.8 | 13 (5.2) | 8 (3) | 2 (7) | 2.71 (0.52-14.14) | 0.24 |
| Tachypnoea (>24 breaths/min) | 10 (4.0) | 7 (3) | 1 (3) | 1.21 (0.14-10.46) | 0.86 |
| Median Oxygen saturation (IQR( | 6 (2.4) | 98 (97-98) | 97 (96-98) | 0.78 (0.62-0.97) | 0.03 |
| Median CRB-65 score (IQR) | 11 (4.4) | 0 (0-1) | 0 (0-1) | 2.05 (0.98-4.29) | 0.06 |
| Blood test results | | | | | |
| CRP (mg/l) | | | | | |
| Median (IQR) | 6 (2) | 5.4 (1.0-14.8) | 24.1 (5.2-81.5) | 1.33 (1.18-1.50)§ | 0.00 |
| >20 | 6 (2) | 48 (20) | 16 (53) | 6.46 (2.88-14.53) | 0.00 |
| >30 | 6 (2) | 40 (17) | 14 (47) | 6.29 (2.75-14.38) | 0.00 |
| >50 | 6 (2) | 22 (9) | 8 (27) | 5.17 (1.95-13.69) | 0.00 |
| >100 | 6 (2) | 8 (3) | 5 (17) | 14.00 (3.16-62.13) | 0.00 |
| Procalcitonin (µg/l): | | | | | |
| Median (IQR) | 6 (2) | 0.05 (0.03-0.07) | 0.07 (0.03-0.12) | 1.34 (0.97-1.83)¶ | 0.07 |
| >0.25 | 6 (2) | 4 (2) | 2 (7) | 7.54 (1.02-55.64) | 0.05 |
| >0.50 | 6 (2) | 2 (1) | 1 (3) | 7.31 (0.45-120.08) | 0.16 |
| Midregional proadrenomedullin (MR-proADM) | | | | | |
| Median (IQR) | 8 (6) | 0.58 (0.47-0.76) | 0.67 (0.53-0.82) | 2.87 (0.73-11.20) | 0.13 |
| >0.646 | 8 (6) | 96 (40) | 17 (57) | 2.19 (1.01-4.74) | 0.05 |
| >1,00 | 8 (6) | 16 (7) | 4 (13) | 2.55 (0.77-8.50) | 0.13 |

§ Per 10 mg/l increase

¶ Per 0.1 µg/l increase

Table S4A: variables used to make the equation for the model with signs and symptoms only

| Diagnostic variable | Multivariable OR (95%CI) | P value | B |
| --- | --- | --- | --- |
| Runny nose absent | 3.13 (1.39-7.06) | 0.01 | 1.14 |
| Feel ill | 12.81 (2.92-56.28) | 0.00 | 2.55 |
| Intercept | -4.492 |  |  |

Table S4B: variables used to make the equation for the model with signs and symptoms and CRP

| Diagnostic variable | Multivariable OR (95%CI) | P value | B |
| --- | --- | --- | --- |
| Runny nose absent | 3.42 (1.44-8.13) | 0.01 | 1.230 |
| Feel ill | 10.78 (2.38-48.89) | 0.00 | 2.378 |
| CRP > 30 mg/l | 4.82 (1.99-11.66) | 0.00 | 1.572 |
| Intercept | -4.797 |  |  |

We did not use current smoker since this represents more likely the type of patients for which chest X ray was deemed necessary.

**The GRACE analysis in the current study cohort**

In a recent evaluation of the GRACE study, in which 5% of 2820 patients with acute cough had pneumonia on chest X ray, the addition of CRP to the clinical prediction rule correctly reclassified 29% of patients (into low, intermediate or high probability of pneumonia). Mostly, patients were reclassified into a lower risk group. Procalcitonin did not add relevant diagnostic information (1).

Here, we present the results of the evaluation of the GRACE derived-model in our cohort of patients. Our results were entered in the multivariate model of the GRACE cohort to assess the value of CRP to improve prediction by calculating the overall reclassification improvement.
The GRACE equation used results from GP physical examination. Since we do not have these data, we made three models using the GRACE algorithm. Firstly, we assume both crackles and diminished breathing sounds present in all patients, secondly, we assume both crackles and diminished breathing sounds absent in all patients and thirdly, we assume both crackles and diminished breathing sound present in the patients with consolidation and absent in the patients without.
Equations using clinical parameters without and with CRP have been used to classify patients in different predefined risk groups and calculate the overall reclassification improvement when adding CRP to the model.

Results

Using GRACE algorithm to assess overall classification improvement with adding CRP, assuming both crackles and diminished vesicular breathing absent, the overall reclassification improvement is 28.8%. Assuming both crackles and diminished vesicular breathing present, the overall reclassification improvement is 4.4%. Assuming both crackles and diminished vesicular breathing sounds present only in patients with pneumonia, the overall reclassification improvement is 21.8%. See GRACE reclassification and overall reclassification calculation (Tables S5A-C).

Discussion

We have used three scenarios to detect the possible differences in findings of the model. The range of overall reclassification improvement is wide (4.4 to 28.8%), but better than using our own model. Overall reclassification improvement in the GRACE model was 29%.
Using GRACE algorithm, reclassification into low or high risk group when adding CRP occurred in 6 to 35% of patients, depending on the scenario assumed. In addition, in two out of three scenarios, the percentage of patients with pneumonia in the low risk group is too high. Therefore, our best case and worst case scenario do not perform well, making our model less valuable for evaluation of the GRACE prediction model.

Table S5A: GRACE reclassification: comparison of diagnostic risk for presence of pneumonia by GRACE diagnostic model with and without addition of measurement of CRP; Scenario assuming both crackles and diminished vesicular breathing absent.

| **Risk according to “symptoms and signs” model (without CRP)** | **Risk according to “symptoms and signs” model plus CRP continuous** | | | | | | | | |
| --- | --- | --- | --- | --- | --- | --- | --- | --- | --- |
|  | **Patients with pneumonia** | | | |  | **Patients without pneumonia** | | | |
|  | **<2.5%** | **2.5-20%** | **>20%** | **Total** |  | **<2.5%** | **2.5-20%** | **>20%** | **Total** |
| <2.5% | 0 | 1 | 0 | 1 |  | 35 | 4 | 0 | *39* |
| 2.5-20% | 5 | 20 | 1 | 26 |  | 73 | 89 | 0 | *162* |
| >20% | 0 | 0 | 0 | 0 |  | 0 | 0 | 1 | *1* |
| Total | 5 | 21 | 1 | 27* |  | 108 | 93 | 1 | 202* |

* In 13 patients temperature is missing, 1 other clinical variable is missing (absence of runny nose). In 6 patients without pneumonia, CRP values are missing.

From the total cohort, 75 have been reclassified correctly and 9 have been reclassified incorrectly. Therefore, the overall result is 66/229=28.8% correctly reclassified patients with adding CRP to the model.

Table S5B: GRACE reclassification: comparison of diagnostic risk for presence of pneumonia by GRACE diagnostic model with and without addition of measurement of CRP; scenario assuming both crackles and diminished vesicular breathing present.

| **Risk according to “symptoms and signs” model (without CRP)** | **Risk according to “symptoms and signs” model plus CRP continuous** | | | | | | | | |
| --- | --- | --- | --- | --- | --- | --- | --- | --- | --- |
|  | **Patients with pneumonia** | | | |  | **Patients without pneumonia** | | | |
|  | **<2.5%** | **2.5-20%** | **>20%** | **Total** |  | **<2.5%** | **2.5-20%** | **>20%** | **Total** |
| <2.5% | 0 | 0 | 0 | 0 |  | 0 | 0 | 0 | *0* |
| 2.5-20% | 0 | 5 | 6 | 11 |  | 0 | 123 | 7 | *130* |
| >20% | 0 | 0 | 16 | 16 |  | 0 | 11 | 61 | *72* |
| Total | 0 | 5 | 22 | 27 |  | 0 | 134 | 68 | 202* |

* In 13 patients temperature is missing, 1 other clinical variable is missing (absence of runny nose). In 6 patients without pneumonia, CRP values are missing.

From the total cohort, 6+11=17 have been reclassified correctly and 7 have been reclassified incorrectly. Therefore, the overall result is 10/229=4.4% correctly reclassified patients with adding CRP to the model.

Table S5C GRACE reclassification: comparison of diagnostic risk for presence of pneumonia by GRACE diagnostic model with and without addition of measurement of CRP; scenario assuming both crackles and diminished vesicular breathing present in patients with confirmed pneumonia.

| Risk according to sign and symptoms without CRP | Risk according to signs and symptoms plus CRP>30mg/l | | | | | | | |
| --- | --- | --- | --- | --- | --- | --- | --- | --- |
|  | Patients with pneumonia | | | | Patients without pneumonia | | | |
|  | **<2,5%** | **2,5%-20%** | **>20%** | **Total** | **<2,5%** | **2,5%-20%** | **>20%** | **Total** |
| **<2,5%** | 0 | 0 | 0 | 0 | 35 | 4 | 0 | 39 |
| **2,5%-20%** | 5 | 6 | 0 | 11 | 73 | 89 | 0 | 162 |
| **>20%** | 0 | 14 | 2 | 16 | 0 | 0 | 1 | 1 |
| **Total** | 5 | 20 | 2 | 27 | 108 | 93 | 1 | 202 |

* In 13 patients temperature is missing, 1 other clinical variable is missing (absence of runny nose). In 6 patients without pneumonia, CRP values are missing.

From the total cohort, 73 have been reclassified correctly and 19+4=23 have been reclassified incorrectly. Therefore, the overall result is 50/229=21.8% correctly reclassified patients with adding CRP to the model.

| Biomarker | Area under the curve (95% confidence interval) |
| --- | --- |
| C-reactive protein | 0.58 (0.48-0.68) |
| Procalcitonin | 0.51 (0.40-0.63) |
| Midregional pro-adrenomedullin | 0.54 (0.43-0.65) |

| Biomarker | Area under the curve (95% confidence interval) |
| --- | --- |
| C-reactive protein | 0.75 (0.65-0.85) |
| Procalcitonin | 0.65 (0.53-0.77) |
| Midregional pro-adrenomedullin | 0.59 (0.49-0.70) |

1. van Vugt SF, Broekhuizen BD, Lammens C, Zuithoff NP, de Jong PA, Coenen S, et al. Use of serum C reactive protein and procalcitonin concentrations in addition to symptoms and signs to predict pneumonia in patients presenting to primary care with acute cough: diagnostic study. BMJ. 2013;346:f2450.
